# Evidence of Factorial Validity of Parental Knowledge, Control and Solicitation, and Adolescent Disclosure Scales: When the Ordered Nature of Likert Scales Matters

**DOI:** 10.3389/fpsyg.2016.00941

**Published:** 2016-06-22

**Authors:** Francesca Lionetti, Loes Keijsers, Antonio Dellagiulia, Massimiliano Pastore

**Affiliations:** ^1^Department of Biological and Experimental Psychology, Queen Mary University of LondonLondon, UK; ^2^Department of Developmental Psychology, Tilburg UniversityTilburg, Netherlands; ^3^Department of Brain and Behavioural Sciences, University of PaviaPavia, Italy; ^4^Department of Psychology, Salesian UniversityRome, Italy; ^5^Department of Developmental and Social Psychology, University of PadovaPadova, Italy

**Keywords:** parental monitoring, adolescent disclosure, Likert scales, confirmatory factor analysis, diagonal weighted least squares

## Abstract

For evaluating monitoring and parent-adolescent communication, a set of scales addressing parental knowledge, control and solicitation, and adolescent disclosure was proposed by Kerr and Stattin (2000). Although these scales have been widely disseminated, their psychometric proprieties have often been found to be unsatisfactory, raising questions about their validity. The current study examines whether their poor psychometric properties, which are mainly attributed to the relatively poor conceptual quality of the items, could have been caused by the use of less-than-optimal analytical estimation methods. A cross-validation approach is used on a sample of 1071 adolescents. Maximum likelihood (ML) is compared with the diagonal weighted least squares (DWLS) method, which is suitable for Likert scales. The results of the DWLS approach lead to a more optimal fit than that obtained using ML estimation. The DWLS methodology may represent a useful option for researchers using these scales because it corrects for their unreliability.

## 1. Introduction

Parental monitoring is a core aspect of family relationships that may help to promote adaptation and prevent youths from going astray. Accordingly, it has received significant attention from developmental psychologists interested in studying adolescent social and emotional development. For instance, differences in the quality of parental monitoring have been linked to adolescent antisocial behavior, delinquency, substance use, deviant relationships, and failure to adhere to medical guidelines (Soenens et al., 2006; Darling et al., 2008; Laird et al., 2008; Smetana, 2008; Keijsers et al., 2009; Kiesner et al., 2009; Racz and McMahon, 2011; Fosco et al., 2012; Tolan et al., 2013). Originally, parental monitoring was perceived as a parent-driven activity (Dishion and McMahon, 1998). However, a significant change in perspective arose with the reinterpretation of Stattin and Kerr, which stresses the adolescents contribution to the degree of parents knowledge and hence the active role of the child in the parent-child relationship (Kerr and Stattin, 2000; Stattin and Kerr, 2000). Within this perspective, children are not considered passive recipients but rather active participants in their interactions with their parents, even when the interactions pertain to sharing or not sharing in the content of the adolescents leisure-time activities, or in other aspects pertaining to contexts in which parents are not present, such as school.

Linked to this theoretical assumption, Kerr and Stattin (2000) propose a set of scales for the assessment of parent-child communication. The assessment is operationalized as follows. Parental knowledge refers to monitoring as prototypical defined; parental control refers to the parents use of rules and restrictions to limit the children abilities to engage in activities without informing the parents; parental solicitation refers to the parents asking their children and/or their children's friends for information; and adolescent disclosure refers to the children spontaneous sharing of information about their activities with their parents. Researchers using these scales have adopted either the whole assessment tool, or have selected a subset of these scales, depending on research aims (Racz and McMahon, 2011; Keijsers, 2015). These easily applicable and widely disseminated self-reports, suitable to be completed by children and/or parents, have garnered the interest of both clinical and developmental psychology research. However, as the number of studies that have employed these tools has increased, concerns regarding the validity of the scales also emerged. Thus, the aim of this paper is to contribute to the study of the psychometric properties of these scales, with a specific focus on the estimation method used in the data analyses.

### 1.1. Psychometric properties of parental monitoring scales

After 15 years of research in the field of parental monitoring, there are more than 600 papers indexed in Scopus, PsychInfo, and Web of Science that cite the revised perspective of monitoring in reviews and empirical studies (Kerr and Stattin, 2000; Stattin and Kerr, 2000). This rapid dissemination of the revised construct has been associated with mixed outcomes regarding the internal consistency and factor structure of the scales developed within the parental monitoring framework, thus raising questions about their validity. For example, in a study of 328 Dutch adolescents, Hawk et al. (2008) reported a poor fit of confirmatory factor analysis (CFA) on parental solicitation and parental control scales, a problem that was resolved by removing some of the original items. In a study of 445 Italian adolescents, Miranda et al. (2011) correlated the residuals of two items from the adolescent disclosure scale to obtain an acceptable fit. The same two items had been previously proposed to be part of a different construct, secrecy, by Frijns et al. (2010). This two-factor version of the original scale of disclosure, which was tested longitudinally on a sample of 309 Dutch adolescents, suggested that the common operationalization of adolescent disclosure incorporates two separate constructs. The two factors, secrecy and disclosure, were subsequently tested in an American sample, and marginally acceptable fits were obtained (Keijsers and Laird, 2014).

A common feature of the above-mentioned studies is the use of the maximum likelihood estimation method (ML) for each of the confirmatory factor analyses performed. The ML estimation method treats Likert scales as interval scales. Conversely, statistical recommendations suggest that more reliable results can be derived if Likert-type variables are analyzed while taking into account their categorically ordered nature in an underlying variable approach (Flora and Curran, 2004; Jamieson, 2004; Yang-Wallentin et al., 2010; Pastore and Lombardi, 2014; Casacci and Pareto, 2015). Thus, it cannot be excluded that low fits and CFA estimation problems reported in the literature, and partially solved by removing items or splitting scales, were, in fact, due to statistical rather than theoretical issues. In the current study, to test the factor structure of the well-known and often-applied scales in parental monitoring, we embrace a more analytical approach.

### 1.2. Underlying variable approach

The Stattin and Kerr scales are five-point Likert scales, ranging from 1 = never, to 5 = always. What is assessed, such as the degree to which a child is willing to disclose information about free time and activities to his/her parent(s), is a continuous latent construct that is measured via ordered categorical response items. Nevertheless, the most common statistical analysis technique used assumes that variables have continuous level measurements, an assumption that is based on the belief that statistically treating ordinal data as interval variables will not greatly distort the relationships among variables and results.

**Figure 1:**
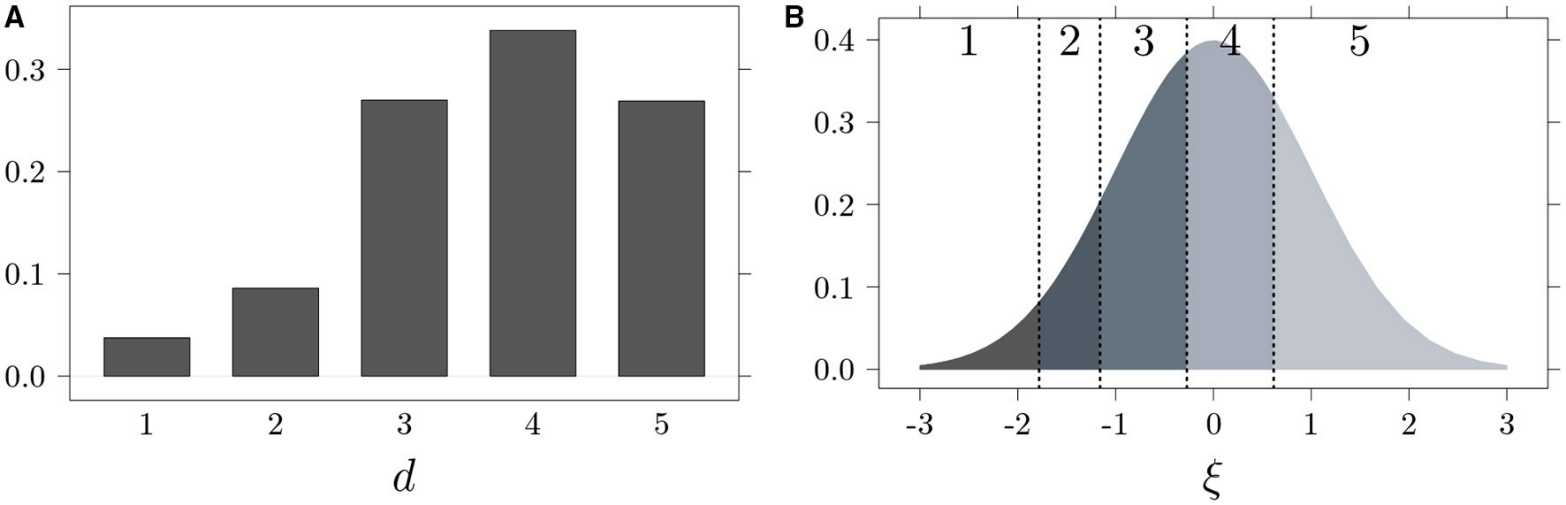
**Likert-type data (A) and related underlying continuous distribution (B)**.

An easily applicable approach for analyzing Likert scales that accounts for their ordinal data nature is the Underlying Variable Approach (UVA; Muthén, 1984; Jöreskog, 1990). Consistent with the UVA, it can be assumed that ordinal item response data *d*, expressed with *k* ordered categories, approximates a latent variable ξ with a normal distribution and a mean equal to 0. Thus, when *d* = *i* (*i*∈{1, …, *k*}), the true value ξ is included between two thresholds, i.e.,:

$$\tau_{i-1}<\xi \leq \tau_{i}$$

where τ_0_ = −∞, τ_1_ < … < τ_*k*−1_ and τ_*k*_ = +∞ are threshold parameters. It follows that given an ordinal item response data with *k* values (e.g., a Likert-type scale with five levels of responses), there are four (i.e., *k* − 1) unknown thresholds. A visual exemplification of how the Likert-type data are related to an underlying continuous distribution is provided in Figure 1. Specifically, Figure 1A represents an item response distribution on a five-point Likert scale, where approximately 4% of the subjects respond 1 to an item, 9% respond 2, 27% respond 3, and 34% respond 4 and 27% respond 5. By using the empirical cumulative distribution function, it is possible to estimate the τ thresholds by applying the formula:

$$\tau_{i}=\Phi^{-1}\left(\frac{\sum_{j=1}^{i}n_{j}}{N}\right)i=1,\ldots ,k-1$$

where *n*_*j*_ is the number of cases in the *j*th category, $N=\overset{k}{\underset{j=1}{\sum }}n_{j}$, and Φ^−1^ is the inverse standard normal distribution function. In panel [B], the underlying normal distribution with thresholds (vertical dotted lines) computed from the discrete distributions depicted in panel [A] is presented.

### 1.3. Aims

In the current study, we aimed to analyze the factorial validity of the parental knowledge, control and solicitation, and adolescent disclosure scales, derived from Kerr and Stattin (2000), testing the scales psychometric properties on a large data set of Italian adolescents. We first evaluated the fit of each separate scale, and we then examined the fit of the theorized structural model—originally tested by Stattin and Kerr within a multiple regression analysis approach—in which parental control, parental solicitation, and adolescent disclosure predict parental knowledge (Kerr and Stattin, 2000; Stattin and Kerr, 2000). Specifically, we explored whether problems originally attributed to the scales, as we have commented in the introduction section, may have been due to the estimation method used in the analysis of the data. In the case of non-satisfactory fit indices, we compared the fit of alternative models derived from the literature.

A core feature characterizing this study is the use of the diagonally weighted least square (DWLS) estimator on a set of scales that are traditionally treated as continuous. The DWLS estimator, which implements UVA and is available in the most widely used software for structural equation modeling, may represent a more reliable option than the popular ML method and avoid estimation biases that can occur with ordered variables (Flora and Curran, 2004; Yang-Wallentin et al., 2010; Pastore and Lombardi, 2014). Also, compared to alternative options for dealing with ordinal variables and non-normal distribution, as the robust ML when continuous observed variables slightly deviate from normality (Rhemtulla et al., 2012), DWLS more explicitly takes into account the ordered nature of categorical variables and may allow avoiding biased results that with the robust ML have been reported with relative small sample size and asymmetric thresholds (Hox et al., 2010; Rhemtulla et al., 2012; Li, 2015). It is the first time, to the best of our knowledge, that the DWLS estimator has been used for testing the factorial structure of the already mentioned scales, i.e., monitoring, knowledge, solicitation, control, and disclosure. Furthermore, it is the first time that the current scales have been analyzed in terms of their psychometric properties on a large data set of Italian adolescents.

## 2. Materials and methods

### 2.1. Participants, procedure, and measures

The participants in this study included 1071 Italian adolescents (28% female) between 13 and 18 years of age (*M* = 16.1, *SD* = 1.23). Upon consent of the adolescents, their parents, and their teachers, paper-based questionnaires were administered individually in a classroom setting to the participants. The four 5-point Likert-scales, originally proposed by Kerr and Stattin (2000), were used.

The parental knowledge (PK) scale consisted of nine items that captured the degree of parents knowledge of their adolescents activities. These items were as follows: Do your parents know what you do during your free time? (item 1); Do your parents know with whom you associate during your free time? (item 2); Do your parents usually know what type of homework you have? (item 3); Do your parents know what you spend your money on? (item 4); Do your parents usually know when you have an exam or paper due at school? (item 5); Do your parents know how you do in different subjects at school? (item 6); Do your parents know where you go when you are out with friends at night? (item 7); Do your parents normally know where you go and what you do after school? (item 8); and In the last month, have your parents ever had no idea where you were at night? (item 9).

The adolescent disclosure (AD) scale consisted of five items that measured the degree of adolescents disclosure of information to their parents. The questions were as follows: Do you spontaneously tell your parents about your friends (the friends you hang out with and their thoughts and feelings on various topics)? (item 10); How often do you usually want to tell your parents about school (regarding, e.g., details about how you are doing in your classes and your relationships with teachers)? (item 11); Do you keep a lot of secrets from your parents about what you do during your free time? (item 12, reversed); Do you like to tell your parents what you do and where you go during your free time and in the evenings evening? (item 13); and Do you keep information about what you do at night and on the weekends from your parents? (item 14, reversed).

The parental solicitation (PS) scale consisted of five items that captured parents tendency to request information from their children. The items were as follows: How often do your parents talk with your friends when they come over to your house? (item 15); How often do your parents ask you about what happened during your free time? (item 16); During the past month, how often have your parents initiated a conversation with you about your free time? (item 17); When did your parents last have extra time to sit down and listen to you when you talk about what happened during your free time? (item 18); and How often do your parents ask you to tell them what happened at school on a regular school day? (item 19).

The parental control (PC) scale consisted of six items that measured the degree to which adolescents are required to inform parents of their activities. The questions were as follows: Must you have your parents permission before you go out on weeknights? (item 20); If you go out on a Saturday evening, must you inform your parents beforehand about with whom you are going and where you are going? (item 21); If you have been out past curfew, do your parents require that you explain why and tell who you were with? (item 22); Do your parents demand that they know where you are in the evenings, who you are going to be with, and what you are going to do? (item 23); Must you ask your parents before you can make plans with friends about what you will do on a Saturday night? (item 24); and Do your parents require that you tell them how you spend your money? (item 25).

### 2.2. Analytic plan

We used a cross-validation approach (Cudeck and Browne, 1983), meaning that we split the original sample into two independent randomly chosen sub-samples, the calibration sample, which included *N*_*c*_ = 643 subjects, and the validation sample, which included *N*_*v*_ = 428 subjects. On the calibration sample, we first explored the item distribution using a visual representation. Second, we performed a series of CFAs for each scale using both the traditional ML estimation method, which is suitable for interval variables, and the DWLS, which implements UVA, as suggested for Likert-type ordinal data (Flora and Curran, 2004). We also tested alternative models, as earlier proposed in the literature, if the fit was not satisfactory. Third, we tested the structural model originally proposed by Stattin and Kerr (2000). Finally, on the validation sample, we re-tested the structural model, and we compared the fit indices and parameter estimates derived from the validation and calibration samples.

For evaluating the similarity between the calibration and validation model estimates, we used the root mean square error (RMSE) based on the following discrepancy measure:

$$\begin{array}{l} \text{RMSE}=F\left({\hat{\theta }}^{v},{\hat{\theta }}^{c}\right)=\underset{i}{\sum }{\left({\hat{\theta }}_{i}^{v}-{\hat{\theta }}_{i}^{c}\right)}^{2} \end{array}$$

in which ${\hat{\theta }}^{v}$ and ${\hat{\theta }}^{c}$ represent the estimated vector parameters into the validation and calibration samples. Additionally, we used the same discrepancy function, modified as follows, for estimating the cross-validation index (CVI; Browne and Cudeck, 1993):

$$\begin{array}{l} \text{CVI}=F\left({\text{S}}_{v},{\hat{\Sigma }}_{c}\right)=\underset{i}{\sum }\underset{j\leq i}{\sum }{\left(s_{ij}-{\hat{\sigma }}_{ij}\right)}^{2} \end{array}$$

in which ${\hat{\Sigma }}_{c}$ is the estimate of the reproduced correlation matrix in calibration sample and **S**_*v*_ the correlation matrix in validation sample.

The data analyses were performed with the R statistical software (R Core Team, 2014) and using lavaan (Rosseel, 2012) and semPlot (Epskamp, 2014) packages.

## 3. Results

### 3.1. Items distribution

**Figure 2:**
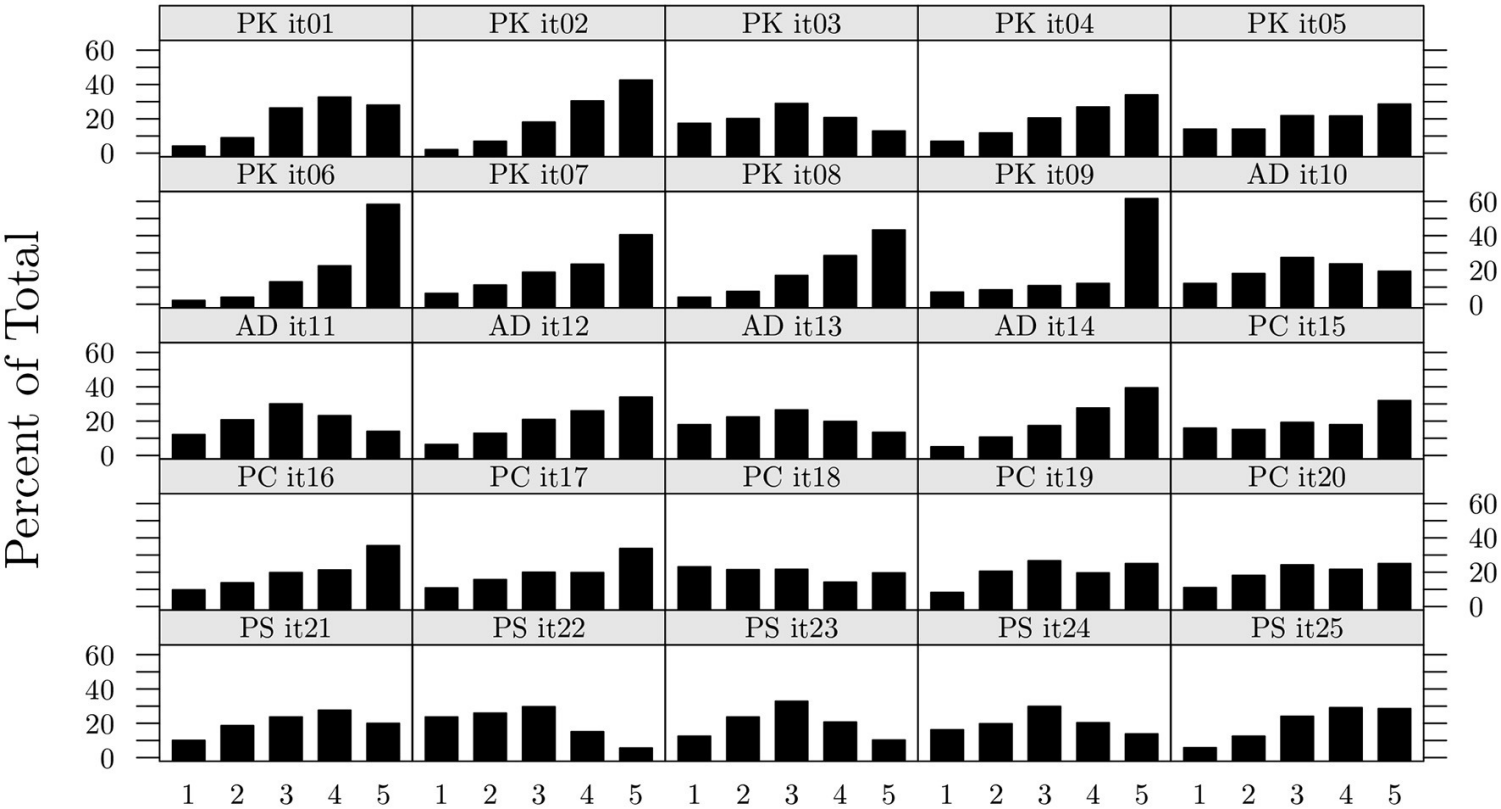
**Item distributions of the four parental monitoring scales (calibration sample ***N***_***c***_ = 643)**.

A graphical representation of item distribution of the calibration sample is displayed in Figure 2. Items 1 to 9 belong to the PK scale, items 10 to 14 belong to the AD scale, items 15 to 20 belong to the PC scale, and items 21 to 25 belong to the PS scale. Most of the items exhibit a skewed distribution.

### 3.2. CFAs of scales

The following calibration sample CFA fit indices are reported in Table 1: comparative fit index (CFI; Bentler, 1989, 1990), non-normel Fit Index (NNFI; Tucker and Lewis, 1973; Bentler and Bonett, 1980), RMSE of approximation (RMSEA; Steiger and Lind, 1980; Steiger, 1989), ML-based standardized root mean square residual (SRMR), and its DWLS-based equivalent, i.e., the WRMR (Weighted Root Mean Square Residual; Muthén, 2004). Also, the total coefficient of determination (TCD; Bollen, 1989) is considered. The incremental measure of fit CFI and NNFI indicate an optimal fit when their values are greater than 0.95; for the absolute measure of fit named RMSEA and SRMR the cut-off suggested are respectively 0.08 and 0.06 (Hu and Bentler, 1999), and for the DWLS-based WRMR, the closer to 1 is, the better the model (Yu, 2002). TCD estimates the amount of explained model variance and ranges from 0 (i.e., 0% of variance explained) to 1 (i.e., 100%) such that the closer it is to 1, the better the fit (Bollen, 1989). On the left side of Table 1 are the indices computed using the ML estimator, whereas the right side lists the indices computed using the DWLS.

**Table 1:** **CFA fit indices for the four scales (***N***_***c***_ = 643)**.

	**ML**					**DWLS**				
	**CFI**	**NNFI**	**RMSEA**	**SRMR**	**TCD**	**CFI**	**NNFI**	**RMSEA**	**WRMR**	**TCD**
ASD	0.67	0.34	0.26	0.13	0.74	0.84	0.68	0.30	3.17	0.77
(a)	1.00	0.99	0.03	0.02	0.74	1.00	1.00	0.03	0.44	0.78
(b)	1.00	0.99	0.03	0.01	0.73	1.00	1.00	0.01	0.31	0.77
PC	0.94	0.90	0.12	0.05	0.85	0.99	0.98	0.09	1.23	0.89
PK	0.88	0.85	0.10	0.06	0.84	0.97	0.96	0.10	1.61	0.88
PS	0.95	0.91	0.10	0.04	0.77	0.99	0.98	0.08	0.90	0.81

*AD, Adolescent Disclosure, (a) two-factor model proposed by Frijns et al. (2010), (b) model with correlated residual variances proposed by Miranda et al. (2011), PC, Parental Control; PK, Parental Knowledge; PS, Parental Solicitation*.

Overall, the DWLS fit indices appear to be better than the ML indices along all scales. This is particularly evident for the PK scale, whose items are among the most skewed (see Figure 2, items 10 to 14). Using the ML method would have led to rejecting the scale, whereas conversely, the fit improved substantially with the DWLS for all indices but RMSEA, which remained mediocre. Only one scale, the AD scale, exhibited overall non-acceptable fit indices for both the ML and the DWLS method. Therefore, we tested the two AD alternative options derived from the literature, and reported in the introduction section, one being a two-factor solution that postulates the existence of two separate components named secrecy and disclosure (see Table 1, model AD(a); Frijns et al., 2010), and the other including correlated errors between the two items included by Frijns and colleagues in the secrecy scales (model AD(b); Miranda et al., 2011). Both options exhibited good fit indices, but only the two-factor solution was used for the structural model data analysis as the two-factor option has received theoretical support from the literature (Frijns et al., 2010).

### 3.3. Structural model

**Figure 3:**
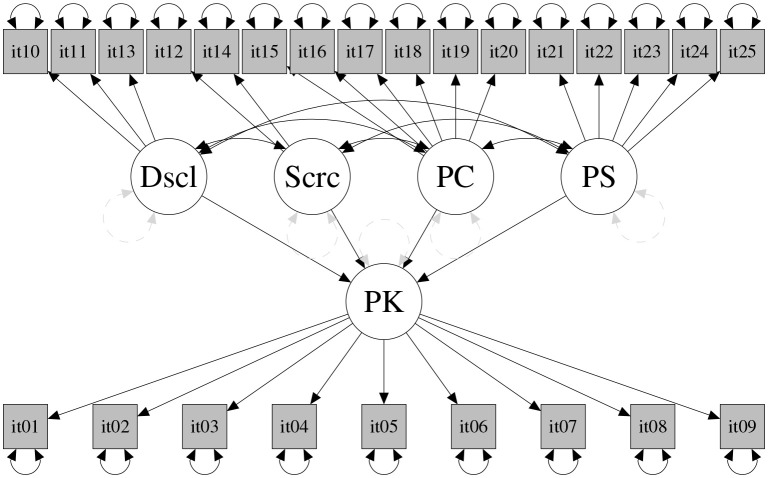
**Structural model**. Dscl, Disclosure; Scrc, Secrecy; PS, Parental Solicitation; PC, Parental Control (PC); PK, Parental Knowledge.

Subsequently, we tested the structural model depicted in Figure 3 with the latent variables parental control, solicitation, and adolescent disclosure as predictors of the latent variable parental knowledge. The model was tested on the validation and the calibration samples. Again, the DWLS fit indices were more satisfying than were the ML indices in both samples (see Table 2); we thus present and comment factor loadings derived from the DWLS method, as those based on ML were rejected for the less than optimal fit indices obtained^1^. In Table 3 are reported factor loadings for the effect of the candidate predictors on parental knowledge, and in Table 4 the correlation among all variables; results were comparable in the calibration and in the validation sample, supporting the reliability of the model tested. Specifically, factor loadings estimated using DWLS ranged from 0.43 to 0.86 for the calibration sample, and from 0.35 to 0.98 for the validation sample. It is worth noting that the values for the calibration and validation samples were similar. The RMSE was 0.706 (*p* = 0.87, estimated by a 3000 bootstrap replicate) and the CVI index was 0.754 (*p* = 0.993, estimated by a 3000 bootstrap replicate). The parameters of the structural model (see Table 2) suggest that the highest contribution to parental knowledge came from the disclosure scale and, to a lesser extent, from parental control and low levels of secrecy. Conversely, parental solicitation was not significantly linked with parental knowledge.

**Table 2:** **Fit indices for structural model depicted in Figure 3**.

		**ML**					**DWLS**				
	***N***	**CFI**	**NNFI**	**RMSEA**	**SRMR**	**TCD**	**CFI**	**NNFI**	**RMSEA**	**WRMR**	**TCD**
Calibration	643	0.85	0.83	0.07	0.06	0.71	0.97	0.97	0.07	1.62	0.74
Validation	428	0.86	0.84	0.07	0.06	0.73	0.98	0.98	0.07	1.39	0.76

**Table 3:** **Factor loadings of the structural model represented in Figure 3, estimated in the calibration and validation samples**.

	**Calibration**					**Validation**				
	**Disclosure**	**Secrecy**	**PC**	**PS**	**PK**	**Disclosure**	**Secrecy**	**PC**	**PS**	**PK**
it01					0.73					0.72
it02					0.66					0.73
it03					0.57					0.67
it04					0.59					0.73
it05					0.64					0.68
it06					0.63					0.65
it07					0.81					0.78
it08					0.73					0.73
it09					0.43					0.37
it10	0.76					0.77				
it11	0.69					0.65				
it12		0.82					0.87			
it13	0.70					0.76				
it14		0.78					0.76			
it15			0.77					0.77		
it16			0.89					0.86		
it17			0.64					0.64		
it18			0.71					0.74		
it19			0.59					0.66		
it20			0.75					0.75		
it21				0.58					0.58	
it22				0.66					0.70	
it23				0.70					0.78	
it24				0.67					0.72	
it25				0.60					0.63	

**Table 4:** **Factor correlations of the structural model represented in Figure 3, estimated in the calibration and validation samples**.

	**Calibration**				**Validation**			
	**Disclosure**	**Secrecy**	**PC**	**PS**	**Disclosure**	**Secrecy**	**PC**	**PS**
Disclosure	1.00				1.00			
Secrecy	−0.38	1.00			−0.41	1.00		
PC	0.43	−0.12	1.00		0.41	−0.15	1.00	
PS	0.68	−0.16	0.49	1.00	0.82	−0.24	0.45	1.00

## 4. Discussion

In the last 15 years, many studies have documented the relationships among parental knowledge, adolescents disclosure, and conduct problems (Racz and McMahon, 2011; Keijsers, 2015). Given the wide dissemination of the results from these studies, it is important that the factorial validity of the scales used to tap into these constructs be carefully examined. However, thus far, mixed outcomes about the scales validity have been reported, and these problems have been solved by deleting some items, correlating items, or splitting the scales into two parts.

In the current paper, using an analytical approach, we proposed that previously reported problems may have been due to statistical rather than theoretical issues. We performed a series of CFA analyses for each scale, testing two different estimators, the ML, which is suitable for interval data and most often used in this field, and the DWLS, which is recommended for ordered categorical variables such as Likert scales (Flora and Curran, 2004). Our results suggest that the DWLS estimator, now available in several statistical software, takes into account the ordered nature of the Likert scales, yielding optimal fits. Specifically, this was true for all scales except the adolescent disclosure scale, whose fits were still relatively poor for the original five-item version. Rather, it was the Frijns and colleagues proposed version (2010), with two factors, disclosure and secrecy, to receive the best support.

Although acceptable fits have been achieved using DWLS, it cannot be excluded that the scales might need to be further revised (e.g., see the adapted self-disclosure scale); also it can be posited that low fit indices previously reported in the literature are due to the absence of a truly latent factor. We propose that it is more likely that items included in the scales represent a subset of all possible issues of which parents may have knowledge (or that children disclose), and thus that is parents overall level of actual knowledge—the latent factor—which causes the scores on the items, rather than the reverse. In support of the existence of specific latent constructs are our results derived from the evaluation of the structural model. Further research in this field may contribute to better disentangle this aspect.

To sum up, conclusions that can be drawn from our study are the following: (1) Stattin and Kerrs scales have acceptable factorial validity, (2) adolescent disclosure may be better (theoretically and statistically) assessed if disclosure and secrecy are considered as separate factors Frijns et al. (2010), and (3) taking into account the ordered nature of Likert scales may lead to more reliable results in the parental monitoring field of research. To explore what this implies from a predictive validity perspective in the field of monitoring, and whether these results apply to different assessment measures based on Likert scales, represent new directions of study in the field of psychological assessment and developmental psychology.

## Ethics statement

The study was carried on in accordance with the Declaration of Helsinki and approved by the institutional review board of the Università Pontificia Salesiana, Rome, Italy.

All participants and their parents received an information sheet under the Italian Law and they were asked to give signed consent by both parents and the participant him/herself.

## Author contributions

FL and LK performed literature review and proposed models to be tested, AD performed data collection, data entry and provides support to the literature review, MP and FL performed statistical analyses and supervised methodological aspects of the paper.

### Conflict of interest statement

The authors declare that the research was conducted in the absence of any commercial or financial relationships that could be construed as a potential conflict of interest.

## Figures and Tables

**Figure A1:**
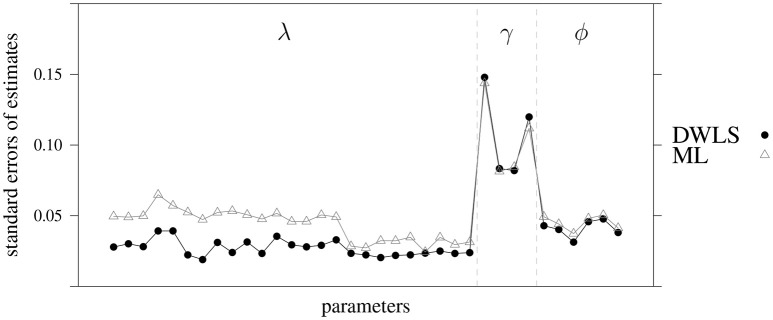
**ML and DWLS standard errors of parameters of the model depicted in Figure 3 and estimated in the validation sample**. Gray triangles refer to ML, black dots to DWLS. λ are factor loadings (see Tables 3, A2), γ structural parameters (see Tables A1, A4), and ϕ the correlation among factors (see Tables 4, A3).

## Footnotes

^1^Parameters estimated using ML are reported in the Appendix. These values, generally lower than DWLS estimates, should be considered with caution given that fit indices identified using ML were not acceptable.

## Appendix

### Parameters estimated by ML

In the following tables are reported parameters of the structural model depicted in Figure 3 and estimated by Maximum Likelihood (ML). Given that ML fit indices of this model were not acceptable, these parameters should be considered with caution. In Figure A1 we depicted standard errors of ML and DWLS estimates, for each parameter of the model estimated in the validation sample. A specific trend emerged: DWLS estimates present overall lower standard errors compared to ML estimates, in particular for λ (i.e., factor loadings). This suggests that the DWLS estimation for the measurement model performed better in agreement with fit indices we identified (see Table 1).

**Table A1:** **Structural parameters (γ, est), standard errors (se), ***z***-values (z), ***p***, and completely standardized estimates (std) for model in Figure 3**.

	**Calibration**					**Validation**				
	**est**	**se**	***z***	***p*-value**	**std**	**est**	**se**	***z***	***p*-value**	**std**
Disclosure	0.78	0.15	5.22	0.000	0.40	1.42	0.26	5.49	0.000	0.67
Secrecy	−0.65	0.09	−6.85	0.000	−0.33	−0.49	0.08	−5.89	0.000	−0.23
PC	0.65	0.09	7.56	0.000	0.33	0.52	0.09	5.86	0.000	0.24
PS	0.20	0.13	1.59	0.112	0.10	−0.08	0.18	−0.45	0.649	−0.04

**Table A2:** **Factor loadings of the structural model represented in Figure 3, estimated in the calibration and validation samples (based on ML estimator)**.

	**Calibration**					**Validation**				
	**Disclosure**	**Secrecy**	**PC**	**PS**	**PK**	**Disclosure**	**Secrecy**	**PC**	**PS**	**PK**
it01					0.68					0.68
it02					0.63					0.71
it03					0.52					0.57
it04					0.59					0.69
it05					0.54					0.59
it06					0.51					0.55
it07					0.75					0.72
it08					0.67					0.70
it09					0.33					0.27
it10	0.73					0.77				
it11	0.60					0.65				
it12		0.86					0.82			
it13	0.69					0.71				
it14		0.67					0.66			
it15			0.75					0.72		
it16			0.78					0.82		
it17			0.60					0.60		
it18			0.69					0.70		
it19			0.48					0.53		
it20			0.71					0.73		
it21				0.48					0.53	
it22				0.70					0.69	
it23				0.66					0.77	
it24				0.66					0.67	
it25				0.53					0.47	

**Table A3:** **Factor correlations of the structural model represented in Figure 3, estimated in the calibration and validation samples (based on ML estimator)**.

	**Calibration**				**Validation**			
	**Disclosure**	**Secrecy**	**PC**	**PS**	**Disclosure**	**Secrecy**	**PC**	**PS**
Disclosure	1.00				1.00			
Secrecy	−0.33	1.00			−0.36	1.00		
PC	0.41	−0.09	1.00		0.40	−0.11	1.00	
PS	0.67	−0.14	0.45	1.00	0.81	−0.19	0.43	1.00

**Table A4:** **Structural parameters (γ, est), standard errors (se), ***z***-values (z), ***p***, and completely standardized estimates (std) for model in Figure 3 (based on ML estimator)**.

	**Calibration**					**Validation**				
	**est**	**se**	***z***	***p*-value**	**std**	**est**	**se**	***z***	***p*-value**	**std**
Disclosure	0.89	0.14	6.21	0.000	0.48	1.28	0.28	4.61	0.000	0.66
Secrecy	−0.51	0.08	−6.25	0.000	−0.27	−0.44	0.10	−4.25	0.000	−0.23
PC	0.62	0.08	7.38	0.000	0.33	0.44	0.10	4.54	0.000	0.23
PS	0.10	0.11	0.93	0.351	0.06	−0.07	0.21	−0.33	0.743	−0.04
